# Bimodal Interventional Instrument Markers for Magnetic Particle Imaging and Magnetic Resonance Imaging—A Proof-of-Concept Study

**DOI:** 10.3390/nano12101758

**Published:** 2022-05-21

**Authors:** Franz Wegner, Kerstin Lüdtke-Buzug, Sjef Cremers, Thomas Friedrich, Malte M. Sieren, Julian Haegele, Martin A. Koch, Emine U. Saritas, Paul Borm, Thorsten M. Buzug, Joerg Barkhausen, Mandy Ahlborg

**Affiliations:** 1 Department of Radiology and Nuclear Medicine, University Hospital Schleswig-Holstein, 23562 Luebeck, Germany; malte.sieren@uksh.de (M.M.S.); joerg.barkhausen@uksh.de (J.B.); 2 Institute of Medical Engineering, University of Luebeck, 23538 Luebeck, Germany; luedtke-buzug@imt.uni-luebeck.de (K.L.-B.); thomas.friedrich@imte.fraunhofer.de (T.F.); koch@imt.uni-luebeck.de (M.A.K.); thorsten.buzug@imte.fraunhofer.de (T.M.B.); mandy.ahlborg@imte.fraunhofer.de (M.A.); 3 Nano4Imaging, 40225 Duesseldorf, Germany; sjefcremers@nano4imaging.com (S.C.); pbo@nano4imaging.com (P.B.); 4 Fraunhofer Research Institution for Individualized and Cell-Based Medical Engineering IMTE, 23562 Luebeck, Germany; 5 Zentrum für Radiologie und Nuklearmedizin, 04103 Dormagen, Germany; haegele.julian@googlemail.com; 6 Department of Electrical and Electronics Engineering, Bilkent University, 06800 Ankara, Turkey; saritas@ee.bilkent.edu.tr; 7 National Magnetic Resonance Research Center (UMRAM), Bilkent University, 06800 Ankara, Turkey

**Keywords:** magnetic particle imaging, magnetic resonance imaging, hybrid imaging, nanoparticles, interventional devices, endovascular interventions

## Abstract

The purpose of this work was to develop instrument markers that are visible in both magnetic particle imaging (MPI) and magnetic resonance imaging (MRI). The instrument markers were based on two different magnetic nanoparticle types (synthesized in-house KLB and commercial Bayoxide E8706). Coatings containing one of both particle types were fabricated and measured with a magnetic particle spectrometer (MPS) to estimate their MPI performance. Coatings based on both particle types were then applied on a segment of a nonmetallic guidewire. Imaging experiments were conducted using a commercial, preclinical MPI scanner and a preclinical 1 tesla MRI system. MPI image reconstruction was performed based on system matrices measured with dried KLB and Bayoxide E8706 coatings. The bimodal markers were clearly visible in both methods. They caused circular signal voids in MRI and areas of high signal intensity in MPI. Both the signal voids as well as the areas of high signal intensity were larger than the real marker size. Images that were reconstructed with a Bayoxide E8706 system matrix did not show sufficient MPI signal. Instrument markers with bimodal visibility are essential for the perspective of monitoring cardiovascular interventions with MPI/MRI hybrid systems.

## 1. Introduction

Magnetic particle imaging (MPI) is a new tomographic modality that visualizes the spatial distribution of magnetic nanoparticles (MNPs) with oscillating magnetic fields [1]. MPI offers a sufficient spatial resolution and a very high temporal resolution. Especially due to its real-time capability as well as the lack of ionizing radiation and nephrotoxic contrast agents, it is very promising for the guidance of endovascular interventions. In the last decade, multiple proof-of-principle studies confirmed this potential by illustrating a variety of application scenarios, e.g., stenosis quantification, balloon angioplasty and stent implantation [2,3,4,5]. Due to the tracer-based imaging principle of MPI, two major phenomena must be kept in mind regarding the monitoring of endovascular interventions: 

First, the majority of clinically established interventional instruments and devices are in principle invisible in MPI as they do not generate sufficient MPI signals [6,7,8]. For those that generate MPI signals, heating becomes a potentially limiting factor for the use in the oscillating magnetic fields of an MPI scanner [9]. As an alternative approach, the device can be filled with a tracer agent and thus becomes delineable [10]. This principle can only be used for (balloon) catheters that have a hollow body. However, for all solid devices, e.g., stents and guidewires, this approach is not applicable. Next to direct visualization, there is the possibility, especially for devices with larger diameters, to render them indirectly visible due to a lack of signal at the instrument’s position inside a tracer-filled volume [10]. However, to achieve an accurate visualization of all kinds of non-signal-generating instruments irrespective of surrounding particles and device size, they can be marked with MNPs. So far, this approach has been established for catheters, guidewires and stents using MNP-containing varnishes [5,11]. 

The second phenomenon that must be considered is the lack of tissue signal in MPI, as only the particle signal is used for the image reconstruction. Consequently, a second modality must be added to achieve any information about the surrounding tissue. Here, the first MPI/MRI [12,13,14] and MPI/CT [15] hybrid imaging approaches have been introduced so far. Due to the lack of ionizing radiation, the combination of MRI and MPI seems to be very promising. Here, a wide range of potential applications becomes possible. In particular, minimally invasive therapies for the liver could benefit from a hybrid imaging scenario. On the one hand, the anatomical information regarding the tumor localization is given by MRI with very high tissue contrast. On the other hand, MPI can be used for accurate instrument tracking and quantitative imaging. In addition, the therapeutic effect of the tumor ablation can be monitored by the means of MPI with heating and viscosity mapping [16,17]. Instruments that are safe and clearly delineable in MRI and MPI are a prerequisite for further pursuing this concept of MPI/MRI hybrid imaging for cardiovascular interventions.

The visualization of medical instruments in both modalities can be achieved by MNP-based marking technologies. In MPI, the superparamagnetic behavior of MNPs with their nonlinear magnetization is the basis for signal generation. In MRI, the iron content of MNPs changes the magnetic field homogeneity of the surrounding medium which leads to signal loss. Thus, MNPs are a versatile tool for visualization in both modalities, whereas the imaging principle and the sensitivity of the modalities differ. For MRI, the first clinically approved guidewires are already available. These guidewires also use MNP markers to guarantee MRI visibility [18].

The purpose of this study was the development of instrument markers that result in comparable apparent marker sizes in both MPI and MRI. Therefore, a modality-specific optimization of the marker is needed. Due to the different imaging characteristics of the two modalities, different types of MNPs that are ideally suited for the respective method and do not interfere with each other should be used.

## 2. Materials and Methods

### 2.1. Coating and Samples

**Figure 1:**
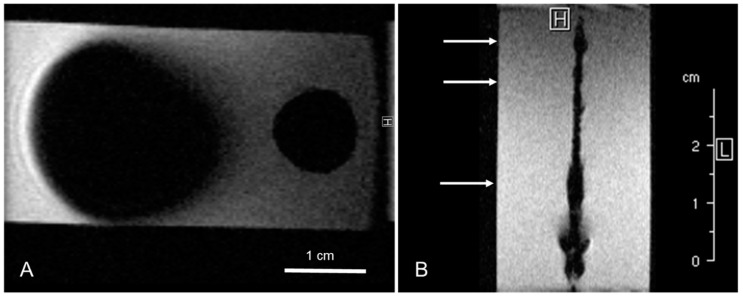
MRI image of varnish samples with a volume of 4 mm³ (**A**) with Bayoxide E8706 (left) and KLB particles (right). The resulting signal voids are comparably larger for Bayoxide E8706 than for KLB. (**B**) A guidewire with three KLB markers in different concentrations (c(Fe) = 2.4 mg/mL, 1 mg/mL and 0.5 mg/mL). In contrast to (**A**), the amount of KLB particles on the guidewire caused no sufficient signal voids at the real marker positions (arrows). The beading at the bottom end is probably caused by contamination of the sample. The B_0_ field direction was perpendicular to the imaging slice.

The markers were synthesized based on two different types of iron oxide particles. For visibility in MPI, synthesized in-house particles (KLB, c(Fe) = 4.8 mg/mL, hydrodynamic diameter: 84 nm) were chosen [19]. For the intended visualization in MRI, commercial particles (Bayoxide E8706, c(Fe) = 200 mg/mL, predominant particle size: 300 nm, LANXESS, Cologne, Germany) were used. The MNP coatings were produced using a specialized coating technology (“Clearcoat”, Nano4Imaging, Duesseldorf, Germany). The method employs two different types of polyurethanes and polyvinyl acetate dissolved in ethyl lactate, which are known to be biocompatible. For testing whether KLB particles generate sufficient signal voids in MRI, two samples (volume: 4 mm³) of Bayoxide E8706 and KLB particles with a coating/MNP ratio of 1:1 and a guidewire with three markers solely containing KLB particles were prepared (Figure 1). 

**Figure 2:**
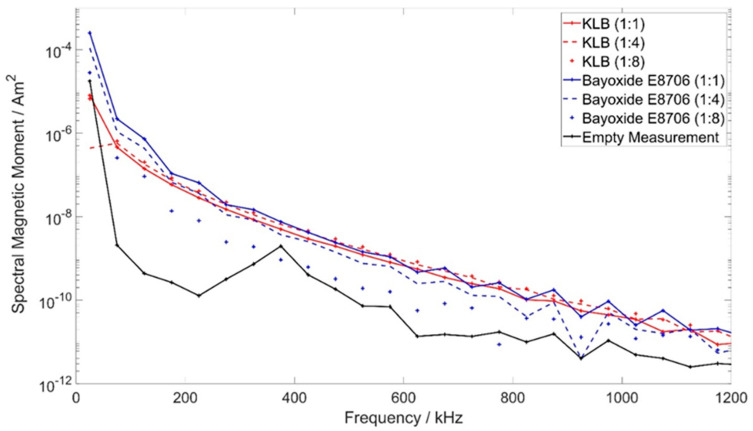
MPS measurements of KLB and Bayoxide E8706 particle coatings.

Based on previously performed magnetic particle spectroscopy (MPS) measurements, which showed sufficient MPI signal of both KLB and Bayoxide E8706 particles (Figure 2) and insufficient MRI properties of KLB particles (Figure 1), the mixing ratio of particles and the coating for the final guidewire was chosen to be 1:1. 

**Figure 3:**
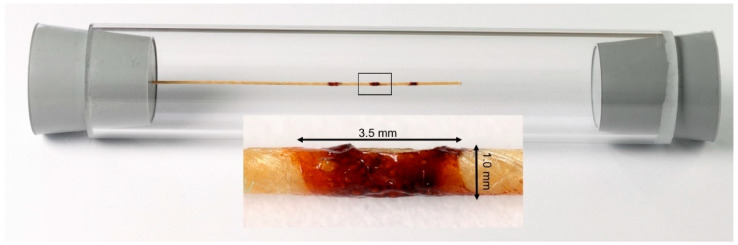
The nonmagnetic guidewire with three discrete markers (distance 10 mm) centrally aligned in an acrylic glass container. The markers contain both KLB and Bayoxide 8706 particles. For the MRI images, the container was filled with diluted gadolinium-based contrast agent. The magnified image of the middle marker illustrates the uneven surface of the coating.

The resulting iron concentration was 2.4 mg/mL of KLB particles and 100 mg/mL of Bayoxide E8706. To place the markers, the coating was applied manually to 10 mm spaced positions on a nonmetallic guidewire segment (Nano4Imaging, Düsseldorf, Germany, material: high-strength core composite of glass fibers and polymers, high-strength aramid fiber mantle) and then air-dried. The resulting marker size was 3.5 mm × 1.0 mm each (Figure 3). 

Before imaging in MRI, the guidewires were dip-coated in impregnating solution (Nanoseal 180W, JELN Imprägnierung GmbH, Schwalmtal, Germany) and cured in an oven (53 °C for 60 min) to protect the markers from being dissolved in the aqueous environment during the experiments.

Since the particles are blended and do not chemically react with each other, no property changes between individual particles and particle blend are expected [20].

### 2.2. Magnetic Particle Spectroscopy

To characterize the MPI signal of the MNP varnish types, MPS measurements were performed in a home-built MPS device [21]. Therefore, 10 µL of the varnish with each particle type was filled in sample tubes with mixing ratios (MNP/clearcoat) of 1:1, 1:4 and 1:8 and air-dried before measuring. The applied excitation frequency was 25 kHz (comparable to the used commercial MPI scanner). The magnetic field amplitude was 20 mT. The measurements were performed with 12,500 repetitions.

### 2.3. MPI Scanner Setup

**Figure 4:**
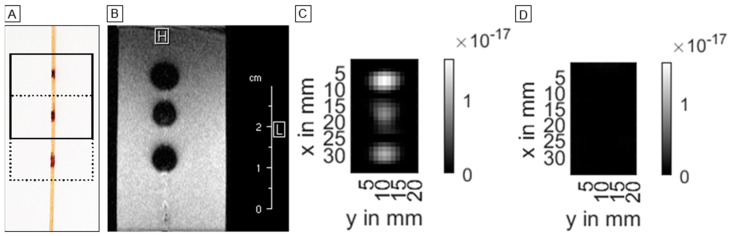
Overview of the marked guidewire (**A**) and the image results in MRI (**B**) and MPI (**C**,**D**). In (**A**), the two positions of the MPI FOV are shown. In MRI, the markers cause circular signal voids (**B**). For MPI, two images are shown: (**C**) shows an image based on the KLB channel of the combined system matrix and (**D**) is the reconstruction result of the Bayoxide E8706 channel (both images showing the central z-slice, i.e., slice 7).

The MPI measurements were performed with a commercial preclinical MPI scanner (MPI 25/20FF, Bruker Biospin, Ettlingen, Germany). The marked guidewire was placed in the center of the scanner’s field of view (FOV) aligned along the *x*-axis on a nonmagnetic home-built phantom holder. The following scan parameters were used: excitation frequencies: 24.5 kHz, 26.0 kHz and 25.3 kHz in x-, y- and z-directions, respectively; excitation field strength: 12 mT in each direction; gradient strength: 2.5 T/m in z-direction and 1.25 T/m in x- and y-directions. The size of the FOV was 19.2 mm × 19.2 mm × 9.6 mm. All measurements were averaged 10 times over 250 repetitions, resulting in a scan duration of approximately 54 s. In MPI, a system matrix acquisition is the basis for image reconstruction. It is possible to distinguish different particle types using different system matrices [22]. Thus, contributions of a certain particle type can be suppressed effectively by selecting a system matrix created for another sufficiently different particle type and combining system matrices of different particle types in one system of equations. Prior to imaging, system matrices of each dry MNP coating were acquired. The point samples had a volume of 2 × 2 × 1 mm³ and following iron concentrations: KLB: 2.4 mg/mL, Bayoxide E8706: 100 mg/mL (= 1:1 mixing ratio). The system matrices were acquired at 17 × 15 × 13 positions in a FOV of 23 mm × 20.3 mm × 13 mm, and each system matrix measurement was averaged 50 times. Because the FOV size did not cover all three markers of the guidewire, the images were acquired at two patch positions (by shifting the sample with a robot), each covering two of the markers (Figure 4A). The patch overlap had a size of 8 voxels resulting in a total FOV size of 26 × 15 × 13 voxels.

### 2.4. MRI Scanner Setup

For the MRI measurements, a preclinical commercial 1T MRI system (Icon, Bruker, Ettlingen, Germany) was used. The guidewire was fixed centrally in a plexiglass tube (inner diameter: 26 mm) (Figure 3). The B_0_ field of the MRI scanner was perpendicular to the guidewire. To detect signal loss caused by the iron of the Bayoxide E8706 particles, we used gadolinium-based contrast agent to reduce T1 of the surrounding medium for improved signal void delineation, in combination with T1-weighted imaging. According to already published measurement protocols, Gadovist (Bayer, Berlin, Germany) in a dilution of 1:200 was chosen [23,24]. The phantom was aligned longitudinally within the scanner. A T1-weighted gradient echo sequence was applied. The imaging parameters were as follows: TR = 60 ms, TE = 8.0 ms, flip angle = 20°, FOV = 4.9 cm × 3.0 cm, matrix = 128 × 128, slice thickness = 0.5 mm. The static measurements were averaged 8 times with a resulting scan duration of approximately 61 s.

### 2.5. Image Reconstruction

For MPI reconstruction of the different particle types, the multi-contrast MPI approach was used [22], i.e., combining both system matrices, the KLB system matrix and the Bayoxide E8706 system matrix, in one system of equations. Image reconstruction was performed with a reconstruction framework developed in-house. To combine the two patches, a joint reconstruction was used [25]. The linear system of equations was set up using a Tikhonov regularization and solved using the Kaczmarz algorithm [26] using 3 iterations and a relative regularization parameter of 0.05. For the measurements, a subtraction with an empty measurement was performed to increase signal quality. For the reconstruction, frequency components between 75 kHz and 625 kHz with a signal-to-noise ratio larger than 3 were selected.

The reconstruction of the MRI data was performed with Paravision software (Bruker BioSpin, Ettlingen, Germany).

## 3. Results

### 3.1. MPS Measurements

Both MNP coating types resulted in spectra above the noise level (empty measurement) (Figure 2). Except for the spectrum of Bayoxide E8706 (1:8), the MPS measurements of all samples resulted in spectra within the same range. For the first odd harmonics, the Bayoxide E8706 samples (coating/MNP ratios 1:1 and 1:4) showed higher signal intensities than KLB. The spectra of the MNP coatings containing Bayoxide E8706 particles showed a small reduction of the signal intensity with decreasing iron concentration. There was no clear observable relation between the different concentrations of KLB particles and the resulting intensities of the spectra.

### 3.2. MPI Images

**Figure 5:**
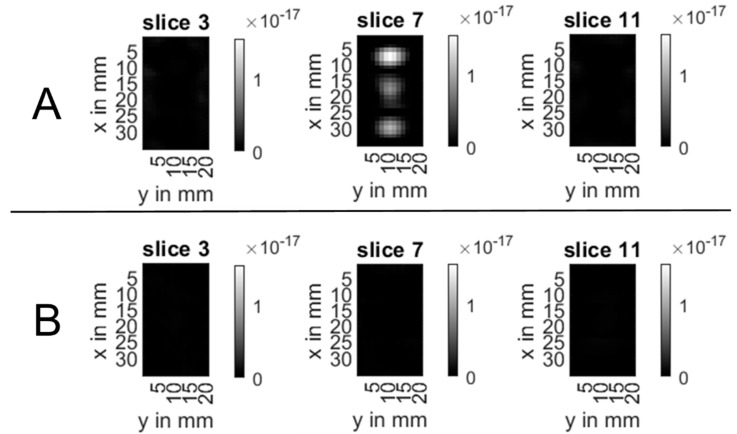
Overview of three representative slices of the reconstructed MPI images. Row (**A**) shows the results for a reconstruction with the KLB channel of the system matrix. The images in (**B**) are based on the Bayoxide E8706 channel of the system matrix with parameters identical to those for the KLB system matrix-based reconstruction.

In MPI, the markers were clearly delineable in the central z-slice (slice 7) as oval regions of high signal intensity in the images that were reconstructed with the KLB channel of the system matrix (Figure 4C and Figure 5A). 

The distal and proximal markers had oval shapes with transversal orientation, whereas the middle marker was aligned longitudinally. The manually measured marker diameters were the following in the xy-planes: 7.0 mm × 11.5 mm (proximal), 11.5 mm × 10.0 mm (middle) and 8.1 mm × 13.0 mm (distal). The displayed marker size was considerably larger than the real marker size. The distance of the markers in the images was around 10 mm, reflecting the real marker spacing (Figure 5). All slices of the KLB reconstructions are scaled to the same intensity range, i.e., the maximum intensity in the volume.

The images which were reconstructed using the Bayoxide E8706 channel of the system matrix did not show any visible MPI signal (Figure 4D and Figure 5B) when all slices were scaled to the same intensity range, i.e., the maximum intensity in the volume. 

### 3.3. MRI Images

**Figure 6:**
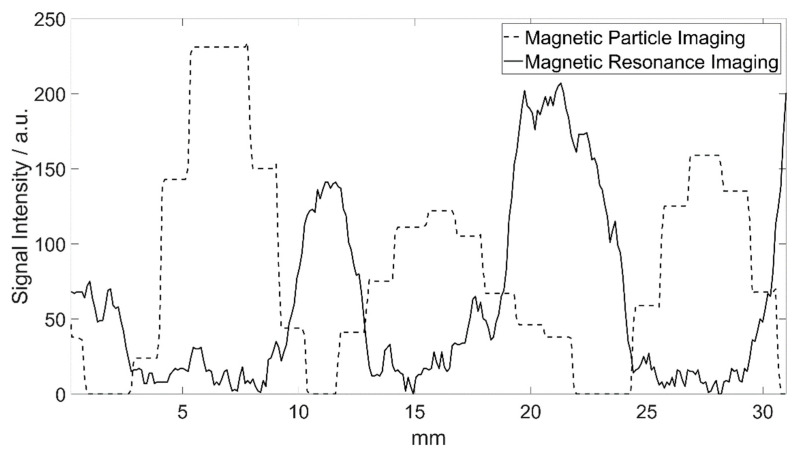
Signal intensity plots of the reconstructed images of both modalities (Figure 4B,C) along the direction of the guidewire. Areas of high signal intensity in MPI are represented as signal loss in MRI. The distances of the maxima/minima of both signal intensity curves correlate very well with the real marker spacing.

In MRI, the markers only containing KLB particles were not clearly delineable from the guidewire’s signal voids (Figure 1). All markers with the combination of Bayoxide E8706 and KLB were clearly distinguishable as circular signal voids (Figure 4B). The maximal marker size was found in the slice directly next to the central slice. The measured marker diameters were: 6.6 mm × 6.0 mm (proximal), 6.2 mm × 5.8 mm (middle) and 6.7 mm × 7.1 mm (distal). The distance between the signal voids’ centers was identical to the distance of the markers on the guidewire (around 10 mm) (Figure 6). The circular signal voids were aligned along a narrow hyperintense band, which seems to be caused by the guidewire (Figure 4B). The signal voids in MRI were located at the positions of high signal intensity in MPI (Figure 6).

## 4. Discussion

In this work, we present markers for interventional instruments based on two particle types that are visible in MPI and MRI. This study illustrates that the presence of different MNPs is a prerequisite for optimal hybrid imaging results and does not negatively influence the depiction of the markers in the respective modality. Furthermore, the resulting marker size in the images is comparable in both imaging methods, despite different particle characteristics and concentrations. 

Today’s gold standard for monitoring cardiovascular interventions is X-ray-based digital subtraction angiography (DSA) and fluoroscopy. In contrast to these methods, in which devices become visible due to their density, in MPI only the particle signal is exploited for image reconstruction. Consequently, most of the established interventional devices are invisible in MPI because they do not generate a sufficient MPI signal [6,7,24]. In recent years, several approaches to overcome this hurdle were presented. Haegele et al. [10] showed the possibility of visualizing balloon catheters by filling them with MNPs or by using the effect of signal voids caused by a water-filled balloon in a phantom full of MNPs. Furthermore, dedicated MPI instrument coatings for catheters, guidewires and stents were presented, and thus a precise visualization of the devices became possible [5,11]. 

It must be acknowledged that due to the use of oscillating magnetic fields in both modalities, a wide range of interventional devices which are used in clinical routine DSA/fluoroscopy are not safe for application in MPI or MRI. Because of the ferromagnetic characteristics and/or the antenna-like shape of the devices, heating is a possible limitation in terms of patient safety [9,27,28]. Nevertheless, MRI-guided interventions became increasingly established over the last few years, and a variety of MRI-compatible instrument designs have been established. The instruments can be tracked by active and passive visualization techniques. Here, it is possible to implement small coils in the devices which allow for active monitoring [29]. Furthermore, the devices can be coated with signal-enhancing (e.g., gadolinium) [30] or susceptibility-affecting materials (e.g., iron oxide) to enable delineation of the instruments in MRI [18,31,32]. In addition, the first human-scaled MPI scanner has been presented, which makes clinical usage of so far preclinical MPI possible [33]. 

To guarantee sufficient visualization of interventional instruments in both modalities, MRI and MPI, a dedicated marking approach becomes necessary. The particles we used for the bimodal markers cause susceptibility artifacts in MRI due to their iron content. However, as the sensitivity of MPI is considerably higher than that of MRI, only a smaller iron concentration of KLB in comparison to Bayoxide E8706 particles is needed for sufficient-quality MPI imaging. The iron concentration of KLB seems to be too small for achieving sufficient susceptibility artifacts in MRI. Despite their significantly higher iron concentration and particle size, the Bayoxide E8706-based coatings showed a comparable MPI signal to KLB in the MPS measurements. Here, the limited validity of the 1D MPS results with respect to the performance of the MNPs under 3D excitation conditions in an MPI scanner must be acknowledged. The reconstructed MPI images with the Bayoxide E8706 channel of the system matrix revealed insufficient reconstruction results. As the optimal particle diameter for MPI is stated to be 30 nm [1], these results might be caused by the finite remanence of the Bayoxide E8706 particles, due to their large diameter, which limits the spatial differentiation of the resulting MPI signal. In principle, a higher remanence should also result in a lower particle response to the oscillating magnetic field. Nevertheless, the high amount of MNPs/iron outside the FOV might have a stronger negative impact on the reconstruction results when using the Bayoxide E8706 channel (c(Fe) = 100 mg/mL) in comparison to the KLB channel of the system matrix (c(Fe) = 2.4 mg/mL). Consequently, the combination of two different particle types can be used to adapt the apparent marker size separately in the two different modalities. Already-presented approaches for bimodal markers used the combination of MNPs for MPI and Cu^2+^ doped water or gadolinium-based contrast agent for MRI [34,35]. In these approaches, the liquid bimodal markers did not share the exact same position. With the coexistence of two particle types within the coating, which is presented in this work, a sufficient co-registration accuracy between both modalities seems to be achievable. In comparison to liquid bimodal fiducials, solid fiducials can guarantee a longer stability and thus can be applied to various surfaces [35]. Furthermore, the solid state is a prerequisite for the introduced application scenario—the visualization of interventional instruments. In addition, it is also possible to extend the use of the marking technology to other devices, e.g., catheters.

Despite all the differences between both imaging modalities, as well as between the two types of particles, the displayed marker size was in a comparable range in each modality. However, the size of the marker images clearly exceeded the true marker dimensions in both methods. As the displayed markers primarily must be clearly distinguishable and guarantee a good visibility in a potential interventional scenario, the overestimation should have no major drawback. However, for an application to smaller vessels, a reduced marker size could become necessary in a possible clinical situation and thus should be investigated in future work. Here, possible approaches could be the reduction of the MNP concentration and a modification of the imaging parameters. It should be kept in mind that higher magnetic field strengths and the orientation of the sample in relation to the B_0_ field of the MRI scanner may influence the apparent marker size.

In this proof-of-principle study, several limitations must be acknowledged: A major limitation is the inconsistency of the marker shape. Due to the high viscosity and the prototype application process, the volume and the morphology of the markers varied. In particular, the application of a defined and reproducible volume during the production of the samples and markers seems to be hampered by the viscosity. Since the focus of our study was to prove the possibility of combining two different particle types, the marker morphology was not addressed. To manufacture reproducible markers, a standardized marking procedure must be developed in the future. Furthermore, the mechanical stability of the markers was not considered in our work. Herz et al. [5] already introduced Resovist-based markers which could withstand the expansion process of an endovascular stent. Especially in the case of vessel bifurcations and curvy vasculature, the markers on the guidewire must resist the resulting mechanical forces. This issue should be part of future studies. Next to the stability, the biocompatibility of the coating should be addressed in upcoming work. Here, the possibility of applying a biocompatible coating to the marked device could overcome possible limitations. In further studies, the particle types should be investigated more thoroughly, both separately and in combination, to obtain deeper knowledge regarding potential, so far undetected interferences. In addition, the imaging results of this in vitro study were acquired in preclinical scanners. Consequently, the results of this work should be confirmed in vivo and must be re-evaluated when human MPI scanners become widely available.

## 5. Conclusions

In this work, interventional instrument markers that can be visualized in both MPI and MRI are presented. This is a basis for designing instruments adapted to vascular interventions guided by MPI/MRI hybrid imaging in the future. It confirms the huge potential of MPI as an emerging vascular imaging technology.

## Data Availability

The data presented in this study are available upon reasonable request from the corresponding author.
